# Tracking Health Data Is Not Enough: A Qualitative Exploration of the Role of Healthcare Partnerships and mHealth Technology to Promote Physical Activity and to Sustain Behavior Change

**DOI:** 10.2196/mhealth.4814

**Published:** 2016-01-20

**Authors:** Sheridan W Miyamoto, Stuart Henderson, Heather M Young, Amit Pande, Jay J Han

**Affiliations:** ^1^ College of Nursing The Pennsylvania State University University Park, CA United States; ^2^ Schools of Health and Clinical and Translational Science Center University of California Davis Sacramento, CA United States; ^3^ Betty Irene Moore School of Nursing University of California Davis Sacramento, CA United States; ^4^ Department of Computer Science University of California Davis Davis, CA United States; ^5^ Department of Physical Medicine and Rehabilitation University of California Davis Sacramento, CA United States

**Keywords:** mHealth, health behavior, motivation, goals, physical activity

## Abstract

**Background:**

Despite the recent explosion of the mobile health (mHealth) industry and consumer acquisition of mHealth tools such as wearable sensors and applications (apps), limited information is known about how this technology can sustain health behavior change and be integrated into health care.

**Objective:**

The objective of the study was to understand potential users’ views of mHealth technology, the role this technology may have in promoting individual activity goals aimed at improving health, and the value of integrating mHealth technology with traditional health care.

**Methods:**

Four focus groups were conducted with adults interested in sharing their views on how mHealth technology could support wellness programs and improve health. Participants (n=30) were enrolled from an employee population at an academic health institution. Qualitative thematic analysis was used to code transcripts and identify overarching themes.

**Results:**

Our findings suggest that tracking health data alone may result in heightened awareness of daily activity, yet may not be sufficient to sustain use of mHealth technology and apps, which often have low reuse rates. Participants suggested that context, meaning, and health care partnerships need to be incorporated to engage and retain users. In addition to these findings, drivers for mHealth technology previously identified in the literature, including integration and control of health data were confirmed in this study.

**Conclusions:**

This study explores ways that mHealth technologies may be used to not only track data, but to encourage sustained engagement to achieve individual health goals. Implications of these findings include recommendations for mHealth technology design and health care partnership models to sustain motivation and engagement, allowing individuals to achieve meaningful behavior change.

## Introduction

### The Field of mHealth Technology

Mobile health (mHealth) technology has captured the attention of health care providers, health system researchers, and the technology industry because of its potential to improve health outcomes, health care services, and health research. The result is an industry that has attracted over US $1.2 billion in venture capital investment in 2014 [1]. Mobile health technologies range from simple text message reminders for health care appointments, to fitness/health apps downloaded for use on mobile phones, to more complex technology that records real-time patient generated data from wearable sensors. Recent research has explored the potential use of mHealth to improve broad health outcomes as well as its utility in specific conditions such as diabetes, heart disease, and cystic fibrosis [2-5]. Despite vast attention paid to this new field, more evidence is needed to understand how this technology can be used, and what health care partners might be involved, to encourage and sustain health behavior change.

### mHealth Technology and Apps

Given that 91% of adults in the United States own a mobile phone [6], 64% of adults own a smartphone, and 15% of smartphone owners report having limited ways to access the Internet outside of their mobile phone [7], mHealth technology has a number of promising apps and possibilities, especially in the areas of health monitoring and health care access. Regarding health monitoring, proponents believe that bidirectional, timely communication of data combined with tailored feedback could play an important role in influencing health behaviors, which may prevent or mitigate factors that lead to disease. For instance, if the technology is connected with pervasive sensors that are either embedded in the environment or on the person, it “can produce continuous streams of data on an individual's biology, psychology (attitudes, cognitions, and emotions), behavior, and daily environment” [8]. With 96% of the US population currently living in areas where mobile networks exist, mHealth supporters also consider the technology to have the potential to improve health care access and reduce health care disparities for hard-to-reach and underserved populations [8]. In a best-case scenario, mobile technology offers the possibility to deliver specialty care where it may not exist, reduce transportation burden, and move care away from traditional clinic and hospital-based care settings, allowing patients to be active participants in the management of their conditions wherever they may be, at times convenient to them [9].

### Motivating Behavior Change

A critical issue for those developing mHealth technologies is the creation of an interface or product that engages the intended user and provides enough value to encourage continued use. A number of studies examining the views of intended consumers of mobile technologies designed to improve health have shown that mobile diaries increased patient’s focus on their disease or their health behavior [3,10,11], mobile displays were effective in encouraging participants to maintain activity levels [12,13], and reminder notifications aimed at goal achievement were desirable features [14]. Evidence also is emerging that indicates text messaging delivered through mobile devices may be beneficial for behavior change, such as smoking cessation, which has implications for other desired health behavior changes that are difficult to undertake and lie within the control of the individual [15]. These studies suggest that customized messaging, feedback, and goal setting are important components of mHealth interventions [2,3,14,16-19].

Research on users of mHealth technology has also identified key design elements such as apps that fit within users’ busy lives [11,20,21], provide personal awareness of activity [11,12,14,18,22], support social networks for sharing, support, or competition [11,12,18,22,23], and provide professional health support [13,18]. Finally, participants in an automated mobile physical activity intervention suggested that individualized coaching that goes beyond messaging prompts was also important [3].

### Sustaining Behavior Change

Prior studies have gathered essential information about the needs and desires of intended users of mHealth technology. Despite these promising findings, however, studies using mobile technology to target health behavior change for physical activity, weight loss, and management of chronic disease have rarely demonstrated long-term effectiveness [24].

To be effective in health behavior change, continuous use of mHealth apps is vital, yet 26% of apps downloaded by consumers are never used a second time [25]. Additionally, 33% of activity tracker owners abandon use of wearable sensors after 6 months [26]. These statistics point to the need for mHealth designers and researchers to focus on how mHealth technology might be used for sustained health behavior change and improved health. The research in this area suggests that behavior changes are more likely to be sustained if patients are involved in identifying and establishing their own goals and include partnerships with health care providers [27].

The broad objective of this study was to further understand potential user’s views of the usefulness of commercially available mHealth technologies to improve individual health and wellness. A more specific aim was to explore sustained use and the role of health care partnerships as users engage with mHealth technology. Sustained behavior change takes time, therefore we sought to determine what elements would encourage ongoing engagement and would assist individuals to make incremental steps in health behavior change and a corresponding improvement in health. Additionally, as a great deal of innovation and consumer health development is occurring without partnership within traditional health care systems, a key point of inquiry was to examine potential mHealth users’ views about including health care partners in their efforts at behavior change.

## Methods

### Setting and Participant Recruitment

This research was initiated to ultimately inform the development of an intervention study (the Wireless Health and Wellness Intervention) focused on improving self-efficacy and exercise health among a health system employee group using mHealth technology. For this reason, we targeted a sample of working adults from an academic institution who were interested in sharing their perspective on what could be offered within an employee wellness program to support sustained health behavior change. The academic health care institution employs over 11,000 faculty and staff.

A convenience sample of focus group participants was recruited through an announcement on the university’s website. Prior to attending the focus group sessions, participants completed a survey that asked for demographic information, past experience with mHealth technology, and self-rated health. Participants were compensated US $50 for their time attending the group. The Institutional Review Board reviewed the study protocol and, as no personal identifiers were collected during the focus group sessions, the research was determined to be exempt from human subjects research.

### Focus Group Data Collection

The research team conducted three focus groups and a final group to confirm study findings (groups included 8-12 participants and the confirmatory group contained three participants). Experienced social science and qualitative researchers, using standard techniques for focus group research, led the focus group sessions [28,29]. Two of the study authors cofacilitated every session. Each session lasted for 1½ hours and was digitally recorded and transcribed. In the beginning of the group session, mHealth was explained and several examples were provided (from pedometers to FitBit, Nike Fuel Band, and other health apps for mobile phones). Participants were asked about their reactions to the mHealth technology, preferences for the type of health data to be collected, potential features or incentives that would sustain their motivation and use of mHealth technology, and their views on sharing data with others, including health coaches or additional health care partners. Although a semistructured interview guide was used, the groups were conducted as “guided conversations”, allowing for frequent probes and unexplored topics to arise [30].

### Data Analysis

The study investigators used a combination of deductive and emergent strategies to identify codes from the focus groups [31,32]. Initially, a review was conducted to identify key issues in the research literature. These ideas, such as privacy, social networking, etc, were used as *a priori* codes. Emergent codes were developed through an iterative process. After the first three focus groups, four members of the research team met and discussed the major issues and topics that arose. These initial topics were used along with the *a priori* codes to conduct an initial independent review and coding of the focus groups. In the review of the transcripts, the researchers found additional ideas that had not previously been coded and the team met again to establish new codes. Continuing this iterative process of coding and review, the research team identified the linkages between the codes and grouped them into broader themes [32].

By the end of the third focus group, the main ideas being introduced in the groups were coalescing around similar themes, such that data saturation was determined to have been met. At that point, one additional small group (n=3) was conducted to validate identified themes and confirm that no additional themes were missed. The validation group was smaller to allow for a more in-depth discussion of the themes than a larger group would have allowed. The coding and analysis process was completed using the qualitative data analysis software program Dedoose [33]. Since conversational analysis was not a goal of the project, in the reporting of quotes, participants’ verbal hesitations and false starts (eg, “umms”) were deleted to improve readability.

## Results

### Participants

The sample of participants was comprised of 24 women and six men (see Table 1). Each group conducted had relative diversity of representation from the various categories (age, income, self-rated health, and use of technology), allowing for balanced perspectives among participant differences. Similar themes were discovered in the three main groups. Participants ranged in age from 25 to 64 years old; with 14/30 (47%) being between the ages 45-54 years old (see Table 1). All participants had some college education with the greatest percentage 13/30 (43%) having attained a bachelor’s degree. Income ranged from US $25,000-$149,999 per year, with the largest number of participants (n=12) reporting earning between $25,000-$49,999. Participants varied in self-reported health ratings. Health rating responses were divided evenly among the “fair”, “good”, and “very good” categories with no respondents rating their health as “poor”, and only 2/30 (7%) rating their health as “excellent” (see Table 2). Over half of the participants 19/30 (63%) stated they were living with at least one chronic condition and 7/30 (23%) reported living with more than one condition. Asthma was the most frequently identified chronic illness 9/30 (30%), followed by obesity 7/30 (23%), arthritis 4/30 (13%), diabetes 3/30 (10%), heart disease, cancer, and prediabetes 1/30 (3% each).

**Table 1:** Demographics of focus group participants (N=30).

Characteristics		Responses n (%)
**Age**		
	25-34	3 (10)
	35-44	6 (20)
	45-54	14 (47)
	55-64	3 (10)
	Missing	4 (13)
**Gender**		
	Male	6 (20)
	Female	24 (80)
**Race/ethnicity**		
	White	19 (63)
	Black or African-American	6 (20)
	Asian	3 (10)
	American Indian or Alaska Native	2 (7)
	Latino/Hispanic/Spanish	4 (13)
	Missing	1 (3)
**Education**		
	Some college, but no degree	8 (27)
	Associate degree	3 (10)
	Bachelor degree	13 (43)
	Graduate or professional degree	6 (20)
**Income (US $)**		
	25,000-49,999	12 (40)
	50,000-74,999	3 (10)
	75,000-99,999	10 (33)
	100,000-124,999	4 (13)

### Participants’ Mobile Technology Use

Regarding their experience with technology, the majority of participants used mobile phone technology 22/30 (73%) and self-identified as being comfortable with technology. Half of the study participants had used a health-related app prior to the focus group 15/30 (50%). All of the participants who attended the group had heard of, or used, a pedometer in the past. Most, however, had not used specialized mobile health activity trackers such as FitBit, Nike Fuel Band, or other devices that measure activity, although they were aware of the technology.

### Drivers for mHealth Technology Use

Participants were introduced to a variety of mHealth technology and were asked to reflect on what factors would encourage or discourage them to use these tools for health improvement. Their initial reactions often focused on the practical dimensions of the technology. Specifically, they considered their daily activities and thought about how the technology might support or augment their current health tracking and monitoring approaches. For example, some participants suggested it was important for the mHealth data to be reliable, such as capturing distance accurately during an exercise session or measuring all of their activities; others emphasized that the device needed to be functional and intuitive. As one participant explained,

I think ease of use completely determines whether I’m going to use it or not because I’ve had lots of apps, and if they’re not easy to use, and not easy to [navigate] and they can’t search…you know, forget it. I’ll stop using it.

Female, age 45-55

Although participants described a wide range of drivers they thought would impact their use of mHealth technology, we focus here on two interdependent, overarching themes—integration and control—that shaped their reflections.

**Table 2:** Technology experience and self-rated health of focus group participants (N=30).

Characteristics		Responses n (%)
**Mobile phone owners**		
	No	8 (27)
	Yes	22 (73)
**Self-reported overall health**		
	Poor	0 (0)
	Fair	8 (27)
	Good	9 (30)
	Very good	11 (37)
	Excellent	2 (7)
**Chronic disease**		
	Yes	19 (63)
	No	11 (37)
**Types of mobile apps used to reach health/wellness goals**		
	Exercise apps	12 (40)
	Improved nutrition apps	9 (30)
	Meditation/stress reduction apps	2 (7)
	Sleep apps	3 (10)
	Haven't used a health-related mobile app	10 (33)
	Other (mood tracker, exercise plan/reminder)	4 (13)
	None	5 (17)

### Integration

The first theme, integration, represents the idea that mHealth technology needs to connect with or complement existing habits and tools. For instance, many participants said the mHealth device should not be redundant with other devices or be something extra that they would have to do. If they already had health tracking tools, they wanted the technology to be integrated with those devices. The most commonly described health tracking tool used by participants was the smartphone. Although not everyone had smartphones in the focus group, the idea of a single integrated device that could capture a range of data in a single platform was appealing.

In addition to wanting data tracking capabilities to be integrated, participants also suggested that data storage and visual display of progress should also be integrated. There was general recognition that the data collected during the course of daily life held value not only as a personal gadget to self-gauge activity, but as an important window into health and overall functioning. Many of the participants had health and medical information collected through a personal health record and thought the integration of health behavior tracking with their health record could provide a more complete picture for themselves and their health care provider. As a participant in one group explained,

[It would be good] to integrate everything together, so, you know, as we get older and have various issues and problems that our physicians have access to information about our exercise, diet, etc, etc and [be able to] bring all that together.

Male, age 50-60

Another participant agreed saying,

And it would just be great to have, in a perfect world, everything all integrated so that my health is a full picture.

(Female, age 35-45

### Control

A second overarching theme in the focus groups was the desire to control the data that would be collected and shared. Some participants wanted to collect an abundance of information such as blood pressure, heart rate, sleep, or mood, whereas others said they wanted minimal information such as calorie intake or energy expended because they would be overwhelmed by too much data.

Look if you give me too much data, you’re just gonna make me crazy. Period. I won’t be able to track it; it wouldn’t do me any good. I’ll get frustrated and I’ll probably [stop using it].

Female, age 40-50

Equally important was the desire to control the type and level of data that would be shared with others, including social network groups or members of their health care team. The majority of participants in the focus group did not want to share their health information through social media networks unless it was shared with a group working toward a common goal that could offer support and motivation. However, without exception, participants thought that the option of sharing their information with health providers was useful. There was some concern, though, as to whether shared data could be potentially available to insurance providers, who might exercise punitive premiums related to negative data. A participant explained,

Right, who has access to the information? Is it protected? I’d like it to be spelled out for me. “No, this cannot be included on any chart that’s going to go to an insurance company or go to whatever.” But, if it’s not clear, then you know, I would probably shy away from [using the technology] until I could be assured that it couldn’t hurt me.

Female, age 40-45

### Sustained Motivation and Engagement: Data Are Not Enough

Beyond the practical features identified by participants as key to encouraging initial interest and use, they discussed how mHealth tools could motivate them to work on health behavior change. In these conversations, it became clear that for most participants, tracking and collecting data were not enough to promote sustained engagement. Participants described needing additional support or structure to help them understand the broader meaning and implications of the data. In discussions of how they might make sense of mHealth data, the importance of context emerged.

### Adding Context

As a participant said, “Unless there’s context to [the data], unless you pull it together and give me some context, I don’t want to sit and figure it out.” Context, as it was talked about in the focus groups, was a sense making activity that included some combination of education and health expertise and could be provided by the mHealth tool or a health care provider.

Context often meant providing information or education that participants were not already aware of, such as whether or how their exercise or sleep habits affect their mood or health. A participant explains how more information would help her place her sleeplessness in context,

Ah, sleep, for me right now, I’ve gotten blind-sided with hot flashes, and you know, I would be really interested to see how often a night I’m woken up by this, how long the hot flash lasts, you know, how it disrupts my sleep because I’m usually a really good sleeper. Then maybe, you know, seeing things that might decrease it. Like if I exercise more.

Female, age 40-50

Some individuals suggested the context could be built into the mHealth device. For example, one participant described wanting an app that could shift her understanding and provide new context to her nutrition habits,

I think it would be cool, if there was a device where we can put on a daily basis like what our calorie intake [is] and then the device could calculate calorie intake with your age, your weight, your physical activity. And let’s say I had 2 bowls of chili for today, and so it will say, “Hey, [name], maybe you should run for 5-1/2 miles to burn whatever calories, you know, that you should burn.”

Female, age 35-45

Although some participants, like the one above, emphasized present context, others wanted the device to provide historical context (ie, to compare their current activity to their past activity). As a male participant explained,

I think for me, I’d much rather have something that could go and ping me when I need it…. Where [the device scans] the data, and it sees that 10,000 steps a day is what my average is and suddenly I’ve got a week of, you know, 7500, 8000. “Hey how are you feeling? What’s going on?” Where I can respond back, “Well, I’ve been sick,” or “Got a big project at work and I’m sitting down...”

Male, age 40-45

### Adding Health Care Partnerships

In addition to discussions about the data not being enough, for some participants, the mHealth devices were not enough to encourage or reinforce behavior change. An active subset of the participants did not want to “figure out” the meaning of their mHealth data on their own. Many were not confident that they would either be able to synthesize the different data elements to understand how it relates to their overall health or that they would have the expertise to know what small changes they might implement to see progress toward their health goals. Noting the limitations of mHealth apps, even those that might be set up to send personalized messages, one participant said,

[A] device could [send]...a simple text like, “Hey, great job on climbing 25 stairs yesterday,” or whatever it may be, you know. So encouragement like that through your device could be useful, but I think to be successful you’re going to have to have some type of interaction with somebody else...because everybody knows kind of what their motivations are, but you also know how easy it is to fall into those pitfalls.

Male, age 30-40

To sidestep potential pitfalls, participants highlighted the importance of interaction, not with the device, but with a health professional to provide context or meaning to the data. As another participant described,

[A health provider] could give me context about oh, this is, you know, the stuff I can’t see maybe, and then kind of tease that out a little bit and say, “Well, you know, you seem to be doing good in this area. How did you feel about how you were doing? But, you know, I notice that your blood pressure has been up or are you taking your meds, or have you been stressed out at work or what’s up?” You know, those kinds of things I think, but absent one or the other, I think there is like a piece missing.

Female, age 50-55

In this understanding of the health care partner’s role, they are not only helping to motivate, but they also provide expertise and help make sense of data. Even more broadly, some saw the inclusion of data as a first step of initiating a “wellness” conversation with a health provider.

I’m tracking all of this stuff, but having somebody say, “Yes, this age group suffers from this. Yes, but this is where you are with it. These are the things that you can do and yes, you’re on track with that.” That confirmation means everything I think in treating one’s wellness....I think everyone looks for that confirmation or the extra knowledge that they are, I use the word on track, that they are doing the right thing to improve their own health.

Female, age 45-55

Some participants raised concerns about whether their providers had the time or interest to review a patient’s personal health goals and the corresponding data, especially given the time constraints of many primary care providers. In discussions of whether the health professional needed to be their primary care physician or whether someone else on the health team could interact with them around the data, participants were open to nurse health coaches, nutritionists, or others who were seen as having expertise in assisting with health self-management. The participants had significant interest in health coaching, which we presented as a partnership where a nurse coach works with someone to set short-term, reasonable, and attainable goals that are patient-centered and monitored over time.

Finally, it is worthwhile to note that not all intended users wanted or needed their data connected to context and meaning. A few participants, for example self-described athletes, were confident they were meeting and often exceeding their own health goals. They reported being highly aware of their activity and health behaviors and suggested that they did not need external context. These participants often were already tracking multiple physical activity performance measures through commercially available sensors or apps on their phone.

## Discussion

### Principal Findings

In our study of potential users of mHealth technology, our findings confirm previous research, which suggests that for potential users of mHealth technology, integrated technology and control are key elements for acceptance and use [3,5,10,11]. Most notable in the discussion for control over data sharing was that there was essentially universal interest in sharing all collected activity data with health care providers. Moving beyond prior research, we also found that study participants envision mobile support that goes well beyond data collection. Our findings suggest that to engage, encourage, and activate individuals to make positive changes in their health behavior, mHealth technology that only tracks and reports data may be insufficient. To maximize the usefulness of the data, focus group participants suggested that the data needed to be placed into context, given meaning, and ideally integrated with their health care data so they can receive individual support and guidance from a health care partner (see Textboxes 3 and 4).

**Patient identified drivers for use of mHealth technology to promote health behavior change.**

1. Integration with:

- Existing technology
- Personal health records

2. Control of:

- Data collected
- Data shared- type, detail, and with whom

**Patient identified needs to sustain use of mHealth technology to promote health behavior change.**

1. Adding context/meaning through:

- Information/Education
- Awareness
- Trends to understand relationship between behavior and outcomes/symptoms

2. Adding health expertise/partnership for

- Data synthesis
- Goal setting for incremental success
- Informed, tailored, regular feedback
- Confirmation of value and importance of patient-centered behavior change work

### Context and Meaning

Context and meaning can develop through multiple avenues. Individuals may find meaning through heightened awareness of their current behavior, through new understanding of how specific behaviors impact their experience of health and wellness, or through partnerships with members of the health care team who can help interpret patient generated data. Participants in this study identified context as a key ingredient for adding value to the data, supporting the theory of “sensemaking”, which highlights that making sense of information requires a process of “acquisition, reflection, and action” [4,34,35]. Sensemaking theory suggests that people use information gathered through their experiences, and if they derive meaning of those experiences in relation to their environment and actions, it allows them to better predict how to make changes and respond to similar situations in the future. Simply collecting patient generated data is only the first stage of the sensemaking process. As our study participants noted, and sensemaking theory suggests, there needs to be opportunities for both reflection and action on information obtained. Often there is a gap in an individual’s ability to synthesize the data and abstract the meaning that may lead to new understanding and ultimately to change. For instance, it may require multiple points of data collection, reflection over time, and consolidated feedback of data to determine if a specific individual sleeps better on days when he or she engages in a vigorous exercise session.

How reflection and action occur will vary depending on the ability of the individual to synthesize and understand how the data relates to their health and behavior. A recent study found that as individuals conduct exploratory causes of changes in health outcomes, they seek affirmation from social networks or their health care provider that they are, in fact, on the right track as they implement changes [36]. If, as many suggested in our focus groups, help is required to synthesize data and create meaning, this could theoretically either be built into device responses or occur in partnership with the individual through collaboration with a member of the health care team. A “patient work” design framework, as outlined by Valdez et al [37], is in alignment with our findings. Specifically, this framework calls for design to include context-sensitive alerts, tailored education, feedback, and personalized settings to support the creation of technologies that are patient-centered [36]. Additionally, we found evidence that some mHealth users may benefit from a “patient-provider” design framework, a design strategy that places the patient-provider relationship (not only the patient) at the center of sensemaking. Potential users not only believed that data gathered from their daily activities were important as they work to make healthy choices, but they also saw the data as a window into their health overall and, therefore, felt it was important for it to be shared with their health care provider. Building on the patient work design, a patient-provider design could have context-sensitive alerts, education, and personalized settings, yet the summary of these interactions and outcomes would be viewable by both patients and health providers so they may share patient-generated health information, leading to collaborative reflection and action.

The patient-provider design is a shared partnership design with foundational elements originally described in the Chronic Care Model [38]. A recent adaptation of the original model, the eHealth Enhanced Chronic Care Model [39], highlights the importance and integration of eHealth components (Internet, social networking, electronic health records, mHealth, and patient portals) into the Chronic Care Model [38]. The elements identified by our participants directly map onto the elements of this model in that they identify a need for education, support, and guidance to be readily accessible to them within their community and outside of traditional office visits. Additionally, participants cited their desire to have the data they generate during their daily activity integrate into their electronic health record specifically so their health care provider could view their activity and provide regular, timely, informed, and personalized feedback to help them reach their goals.

### Limitations

This research was exploratory and had several limitations. One of the limitations was that we asked participants to speculate on what they wanted from mHealth technology. We did not have a mobile health platform for them to test, so they may have had different reactions to the technology if they were interacting with it. We also were not able to test whether their understanding of motivation would in fact motivate them to use the mHealth technology. In future studies, we will interview individuals who have used a specific technology during and after the intervention to get a more complete understanding of how mHealth might be able to help with behavior change.

A second limitation relates to the sample of respondents participating in this study. Participants were selected from a convenience sample of health system employees who responded to a study advertisement. The participants in the focus groups likely had a higher education, were more familiar with the health care environment, were more comfortable with technology, and had higher income than would be expected in a general population. Similar to other studies related to physical activity, more women participated than did men [39-42]. In our focus groups, both women and men suggested the desire for context and health care partnerships. If more men were recruited for the focus groups, we may have seen some variation in the types or levels of partnerships they desired as compared to women. Additional research is needed to explore the generalizability of our findings to other populations, especially if mHealth technology will be used in older populations, lower income groups, or populations less experienced with mobile technology.

### Design Considerations

The findings from this study related to integration suggest the need for consolidated information in one easy-to-access location, with meaningful data points that correlate to other measures of health. The most often identified solution by participants was to link mHealth data with patient portals or personal health records connected to their health system. Issues of control were considered satisfied if sharing rules could be set by the user related to which data elements were shared with each person in their network. When considering the exchange of data with a member of the health care team, there was less concern about sharing all relevant data gathered as long as there was assurance there would not be punitive insurance premiums for “bad data”. Use and sharing of data would need to be defined and made clear to users joining programs where data are incorporated or shared with their health system to alleviate those concerns.

If the findings from this study are to be incorporated into a mHealth intervention design aimed at behavior change, there are a number of challenges. Bringing patient-generated data into closed electronic health record systems presents significant security and system challenges, which must be addressed to provide the desired level of integration identified in our findings. Determining how best to distill big data streams to the right-sized, meaningful summary data that are actionable and can be readily available to both patients and clinicians is also essential.

In addition to technical challenges, there are other clinical considerations that must be explored. It is imperative to determine whether providers believe patient generated data will augment patient care, and if so, what data are important, how much they would like to see (summary vs granular), how the data should be displayed, and how this additional information would fit within current patient care workflow. Importantly, as the above challenges are considered and solutions are found, we must determine how to evaluate the impact of the tools and system integration on the patient experience and outcomes.

Finally, it is important to acknowledge that patients are only one part of the equation in the use of mHealth. Development of mHealth technologies requires an iterative process of obtaining information and guidance from key stakeholders (patients, information technology specialists, and providers), identifying and potentially repurposing existing technology, designing user-friendly systems and taxonomies for data collection and analysis, integrating with health system interfaces, and testing through interventions or randomized trials. Additional structural factors also would need attention, including deciding which health care activities are tied to reimbursement, how health care providers are educated to interact with patients and with other members of the health care team, the culture of medicine and health care (for example, the attention to wellness vs disease), the mode and/or location of health care delivery, and liability issues associated with receiving patient-generated data that might require timely provider action.

### Conclusions

mHealth technology to manage chronic conditions and improve wellness is becoming ubiquitous. Yet this development is occurring in large part independent of the health care system. This study contributes to our understanding of the desired technology and health care elements identified by potential users, which can bridge the gap between personal technology development and integration with the health care system.

Improved health outcomes are essential to encourage health care providers, health systems, insurers, and funders to invest in and adopt novel mHealth technologies on a broader scale. Measurable outcomes will likely require sustained behavior change from both patients and health providers. Because of this, a better understanding of the underlying motivation of intended users of mHealth technology and health providers is essential for intervention success. We must recognize, and address, through programmatic and technological design, the drivers and barriers to technology engagement so that we ultimately offer users the elements to support and motivate them for the difficult task of behavior change. At a basic level, the mHealth tools offered to users should be intuitive, integrate with existing technology, and should allow the user to control what data are collected and shared. At a deeper level, the importance of linking data with context and meaning, suggests that mHealth technology should ideally provide the user with some new understanding of how their behavior affects their health. Finally, to accomplish the goal of providing a fuller picture of health, integrating daily health activities into the clinical record can offer an opportunity for clinical reinforcement of health behavior change.

## Abbreviations

mHealth: mobile health
